# Genetic counseling and diagnostic guidelines for couples with infertility and/or recurrent miscarriage

**DOI:** 10.1515/medgen-2021-2051

**Published:** 2021-05-14

**Authors:** Margot J. Wyrwoll, Sabine Rudnik-Schöneborn, Frank Tüttelmann

**Affiliations:** Institute of Reproductive Genetics, University of Münster, Vesaliusweg 12-14, 48149Münster, Germany; Institute of Human Genetics, Medical University Innsbruck, 6020Innsbruck, Austria

**Keywords:** female infertility, male infertility, miscarriages, genetic counseling, ART

## Abstract

Around 10–15 % of all couples are infertile, rendering infertility a widespread disease. Male and female causes contribute equally to infertility, and, depending on the definition, roughly 1 % to 5 % of all couples experience recurrent miscarriages. In German-speaking countries, recommendations for infertile couples and couples with recurrent miscarriages are published as consensus-based (S2k) Guidelines by the “Arbeitsgemeinschaft der Wissenschaftlichen Medizinischen Fachgesellschaften” (AWMF). This article summarizes the current recommendations with regard to genetic counseling and diagnostics.

Prior to genetic counseling, the infertile couple must undergo a gynecological/andrological examination, which includes anamnesis, hormonal profiling, physical examination and genital ultrasound. Women should be examined for the presence of hyperandrogenemia. Men must further undergo a semen analysis. Based on the overall results, hyper- or hypogonadotropic hypogonadism can be diagnosed in both sexes.

Female genetic diagnostics for infertility comprise karyotyping, analysis of the *FMR1* premutation and a gene panel including genes associated with congenital hypogonadotropic hypogonadism (CHH) or congenital adrenal hyperplasia. Male genetic diagnostics for infertility comprise karyotyping, screening for AZF microdeletions, *CFTR* analysis and a gene panel including genes associated with CHH. Also, gene panels are increasingly being used to causally clarify specific phenotypes such as defective sperm morphology/motility or azoospermia. As infertile couples have an increased risk for chromosomal aberrations, a chromosomal analysis should also be offered to both partners prior to undergoing assisted reproductive technology. In couples with recurrent miscarriages, karyotyping is recommended to detect balanced structural chromosomal aberrations.

## Introduction

A large proportion of genetic consultation appointments is attributed to infertile couples and couples with recurrent miscarriages. Infertility, which is defined by the WHO as the inability to achieve a pregnancy after one year of unprotected intercourse [1], affects 10–15 % of all couples, thus rendering infertility a widespread disease, comparable to, e. g., high blood pressure or depression. Historically, the female partner has been the focus of diagnostics and counseling, but the causes of infertility are well known to be equally distributed between the male and female partners. In fact, in one-third of all infertility cases, both partners contribute to the pathogenesis [2]. Since 5–10 % of female infertility cases and 5–20 % of male infertility cases have identifiable genetic roots in currently established diagnostic analyses [3], [4], such diagnostics and respective counseling should account for both partners equally. Due to novel technologies such as next generation sequencing (NGS) and an increasing number of known monogenic causes of both male and female infertility, more diagnostics increase the complexity of genetic counseling and informed consent for those couples.

In contrast to couples who present with infertility, couples with recurrent miscarriages regularly do not have any difficulty achieving a pregnancy but fail to maintain it. Overall, miscarriages occur frequently, as 5 % of all couples of reproductive age experience recurrent (at least two) miscarriages [5]. The percentage of couples meeting the WHO definition of recurrent miscarriage (three or more miscarriages before 20 weeks gestation) is estimated to be 1 % [3]. Most miscarriages take place within the first trimester and are the result of numerical chromosomal aberrations. In most cases, the fetus is not further examined, leaving the specific cause undetermined.

Although both infertility and miscarriages are common, these issues remain socially taboo. Therefore, concerned couples often feel exclusively affected by this problem and avoid discussing the topic with their peers, which puts these couples under additional pressure; therefore, genetic counseling of infertile couples or couples with miscarriages also requires an associated educational component. On top of that, affected couples often suffer enormous psychological stress. Furthermore, the age of the female partner constitutes a critical factor for conceiving a child naturally. Notably, the average age of women receiving treatments at fertility centers in Europe is 35 years [6], resulting in lower pregnancy and higher miscarriage rates when compared with the normal fertile population.

This review provides an overview of important genetic conditions and the recommended diagnostic algorithms for couples with recurrent miscarriages or infertile couples requesting assisted reproductive technology (ART). The corresponding recommendations were published in German-speaking countries as S2k Guidelines by the “Arbeitsgemeinschaft der Wissenschaftlichen Medizinischen Fachgesellschaften” (AWMF) [4], [7]. For a more in-depth overview, both guidelines have been summarized in articles concerning the female [8], [9], [10] and male [10], [11] considerations.

## Gynecological examinations for reduced fertility

Diagnostic algorithms generally start with determining the patient’s personal disease history including pedigree information (anamnesis) followed by clinical and laboratory examinations. Anamnesis and physical examinations should address pubertal development, signs of hypogonadism or cortisol excess. Anomalies of the inner genital tract can be sufficiently detected by a vaginal sonography.

The endocrine basics include measuring LH, FSH, TSH, prolactin, testosterone, DHEAS, SHBG, free androgen index, estradiol and Anti-Müllerian hormone (AMH). If there are anovulatory cycles, a single progesterone measurement should be obtained at about day 21 in an assumed cycle. If there are abnormal findings in the endocrine basics, further diagnostic steps proceed, e. g., 17-OH progesterone (17-OHP) measurements in the presence of hyperandrogenemia.

### Female hyper- and hypogonadotropic hypogonadism

For genetic testing it is important to distinguish hyper- and hypogonadotropic hypogonadism. Hypergonadotropic hypogonadism is characterized by elevated gonadotropins (FSH/LH) in the presence of normal or reduced estrogen levels and point toward a primary ovarian dysfunction. Hypogonadotropic or hypothalamic hypogonadism (reduced FSH/LH and reduced estrogen levels) is very rare in females and is mostly caused by a reduced secretion of the gonadotropin releasing hormones (GnRH). Genetic causes are complex and include congenital hypogonadotropic hypogonadism (CHH) and metabolic dysfunctions of the pituitary gland.

### Hyperandrogenemia

Important differential diagnoses of hyperandrogenemia are polycystic ovary syndrome (PCOS) and congenital adrenal hyperplasia (CAH). Women with PCOS present with cycle abnormalities and clinical signs of hyperandrogenemia. Following the Rotterdam criteria, the diagnosis of PCOS can be established if testosterone levels are increased and SHBG levels are reduced, along with specific ultrasound changes of the ovaries (more than 12 antral follicles of less than 10 mm in diameter in each ovary). More than 50 % of patients show an insulin resistance, which can best be detected with the HOMA index.

If hyperandrogenemia is associated with high 17-OHP and DHEAS, this points toward an adrenal androgen production as seen in CAH. Classical CAH, most often caused by severe biallelic mutations of the *CYP21A2* gene, is nowadays generally detected by newborn screening for early treatment. Non-classical or late-onset CAH presents like PCOS, with cycle abnormalities, subfertility and hyperandrogenemia. The most frequent 21-hydroxylase deficiency is suspected in the case of elevated 17-OHP and relative reduction of cortisol. Final diagnosis is generally established by molecular genetic analysis.

### Anti-Müllerian hormone

AMH is synthesized by primordial follicles and plays a role in determining ovarian activity. The activity of AMH is inversely correlated with age and shows considerable interindividual differences. Reduced AMH levels are of poor predictive value regarding fertility but are used to estimate the ovarian reserve in the context of ART. Besides genetic factors leading to primary or premature ovarian insufficiency (POI), no genetic conditions resulting in specifically low AMH levels are known.

## Andrological examinations for reduced fertility

Andrological examinations include personal and familial anamnesis, a physical examination, hormonal profiling, exclusion of malformations of the urogenital system, scrotal ultrasound and semen analysis. According to WHO guidelines [12], the semen analysis should not only contain sperm count and sperm concentration but should also assess the morphology and motility of the sperm (Table 1). Based on these findings, clinicians can diagnose oligozoospermia (reduced sperm count), asthenozoospermia (reduced sperm motility) and teratozoospermia (aberrantly shaped sperm). Frequently, combinations of the three conditions are observed, which is then called oligoasthenoteratozoospermia (OAT).

**Table 1:** WHO reference values for semen analysis [12].

Sperm concentration	>15×10^6^/ml
Sperm count	>39×10^6^
Motility	>32 % progressive motile
Morphology	>4 % normal forms
Living sperm	>58 %
Leukocytes	<1×10^6^/ml
Mixed-antiglobulin-reaction (MAR) test	<50 % with adherent particles

### Obstructive vs. non-obstructive azoospermia

The most severe male infertility phenotype, which can be diagnosed through semen analysis, is azoospermia. This term implies that even after centrifugation, no sperm are found in the semen. Azoospermia can have several different etiologies: The most clinically relevant distinction is between cases in which no mature sperm are produced in the testes and cases in which an obstruction prevents the sperm from appearing in the semen. The first condition is called non-obstructive azoospermia (NOA); its phenotype is closely related to cryptozoospermia, i. e., sperm can only be detected after centrifugation of the sample. Azoospermia and cryptozoospermia often form a continuum and should, therefore, receive the same diagnostic procedure. As a rule, NOA can be suspected in azoospermic men with increased FSH levels and reduced testicular volume (<12 ml each).

The second condition is obstructive azoospermia (OA), which may be assumed in azoospermic men with normal FSH levels and normal testicular volume. OA is indicated by additional semen parameters, e. g., reduced values for ejaculate volume, pH and fructose and glucosidase levels. However, these parameters are not regularly determined in all cases.

### Male hyper- and hypogonadotropic hypogonadism

Patients with syndromic male infertility are often already clinically diagnosed prior to genetic counseling because of their hormonal parameters, physical features and delayed or absent puberty. Distinctions between hypergonadotropic and hypogonadotropic hypogonadism can be made using the concentrations of the gonadotropins LH and FSH. If increased values for LH and, in particular, FSH coincide with reduced testosterone levels, a testicular impairment is likely, resulting in hypergonadotropic hypogonadism, a typical finding in Klinefelter syndrome. Conversely, men with hypogonadotropic hypogonadism present with low values for LH and FSH, resulting in reduced levels of testosterone. These findings rather imply an impairment of the hypothalamus or pituitary axis and point towards CHH. Frequently, affected men have experienced a delayed puberty or no puberty at all. If this condition coincides with an inability to smell (anosmia), a subform of CHH, namely Kallmann syndrome, should be suspected.

## Genetic diagnostics in women

**Figure 1:**
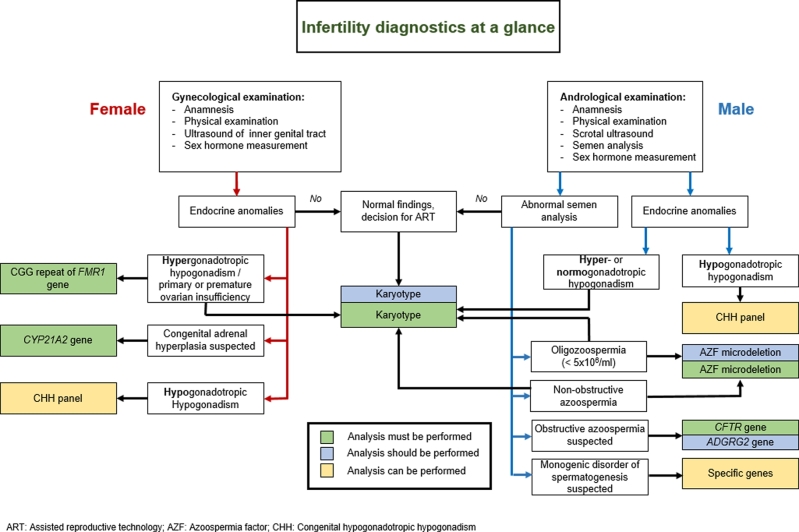
Diagnostic procedures in infertile couples, figure modified from the AWMF guideline 015/085.

Following a gynecological examination that includes hormonal assessments, genetic algorithms can be determined (Fig. 1). About 40 % of women with fertility issues experience cycle abnormalities, pointing toward an ovarian dysfunction; yet, not all of them have clear-cut evidence of hypergonadotropic hypogonadism, which may develop over time. Karyotyping reveals a gonosomal aberration in 10–13 % of women with ovarian dysfunction, such as Turner syndrome (45,X, structurally abnormal X chromosomes or 46,XX/45,X mosaicism) or trisomy X (47,XXX). The knowledge of an abnormal karyotype is important for further management, as women with primary amenorrhea due to Turner syndrome have only little chance of successful ART. Women with trisomy X have largely normal fertility but may develop POI.

In women with primary or secondary POI and a normal karyotype, analysis of the *FMR1* premutation must be performed. Premutations of the *FMR1* gene are associated with an increased risk for fragile X syndrome in offspring. Detection rates are 2 % in single cases without family history and 10–15 % in familial cases of Caucasian females with POI [13]. Depending on the number of CGG repeats of the *FMR1* gene, the risk of a repeat expansion to a full mutation in a child increases dramatically in premutation carriers.

CHH is only rarely encountered in female infertility and affects about 1 in 30,000–40,000 women. Currently more than 20 genes are known to be associated with CHH. Genetic testing (based on gene panels) can disclose the underlying cause and mode of inheritance.

If endocrine assessments give evidence of CAH, molecular genetic testing of the underlying genes is indicated. In more than 90 % of patients, CAH is caused by biallelic mutations of the *CYP21A2* gene, which results in 21-hydroxylase deficiency. If a severe *CYP21A2* mutation is detected in the female partner, genetic testing is indicated in the male partner. If he is also the carrier of a severe mutation, the couple should be advised that prenatal treatment can be offered, to reduce virilization of female fetuses, though this is considered experimental [14]. The corresponding German AWMF Guideline “Adrenogenitales Syndrom mit 21-Hydroxylasedefekt (AGS), pränatale Therapie” (AWMF 174-013) is currently under revision.

## Genetic diagnostics in men

Men with azoospermia, severe oligozoospermia or hypergonadotropic hypogonadism must be offered a chromosomal analysis.

The most frequent chromosomal aberration leading to azoospermia or (rarely) to severe oligozoospermia is Klinefelter syndrome (47,XXY), which in the normal population is found in around 1 of 500 men, while in men with azoospermia it is found in 15 % of men. Generally, men with Klinefelter syndrome present with elevated levels of gonadotropins, strongly reduced testicular volume of at best 5 ml and azoospermia [15]. The correct cytogenetic diagnosis has direct therapeutic consequences for the patient, as it is usually followed by a testicular biopsy with the aim of testicular sperm extraction (TESE); testosterone replacement therapy should not be started prior to the surgery. Although men with Klinefelter syndrome were for a long time regarded as infertile, the reported success rate of sperm retrieval in these men is around 50 % [16]. TESE chances seem to be comparable in adolescents, young adults and adults. Among men with reduced sperm count, the 47,XXY cell line can also be present in a mosaic condition, hampering the clinical diagnosis and allowing no prediction of testicular function and fertility.

Other sex chromosomal aberrations such as the karyotype 46,XX in phenotypic males or a derivative Y chromosome are rare causes for azoospermia. In most 46,XX males, a translocation of the *SRY* locus can be detected, though also monogenic causes are known. A derivative Y chromosome is most commonly seen as a ring chromosome or an isochromosome and warrants analysis concerning the presence or absence of the azoospermia factor (AZF) loci. Balanced structural chromosomal anomalies of the autosomes can also be associated with reduced spermatogenesis.

### Y-chromosomal azoospermia factor microdeletions

In the case of a normal male karyotype, screening for microdeletions on the Y chromosome in the AZF region must be offered to men with azoospermia or severe oligozoospermia. The current practice is to use a sperm concentration of 5×10^6^/ml as a threshold for AZF deletion analyses [17]. Recently, a lower threshold of 1×10^6^/ml has been proposed, as men with higher sperm concentrations only rarely carry complete Y-chromosomal microdeletions [18]. The AZF region comprises three different loci: AZFa, AZFb and AZFc. A complete deletion of any of these loci leads to severely impaired or absent spermatogenesis. Thereby, chances of sperm retrieval by TESE are best in carriers of an AZFc deletion (around 50 %), whereas complete AZFa, AZFb and AZFbc deletions have a very poor prognosis for TESE (virtually zero).

### Genetic diagnostics in men with obstructive azoospermia

When OA is suspected, analysis of the *CFTR* gene must be performed. Congenital bilateral absence of the vas deferens (CBAVD) is a subform of mild cystic fibrosis. CBAVD is characterized by azoospermia with normal gonadotropins and normal testicular volumes [19]. In contrast to patients with clinically manifesting cystic fibrosis, men with CBAVD most commonly have a severe mutation on one *CFTR* allele and a mild mutation on the second allele. The detection of biallelic *CFTR* mutations predicts a high chance of sperm retrieval by TESE, at nearly 100 %. Preferably before TESE is attempted, the patient/couple must be informed about an increased risk of classical cystic fibrosis in their offspring. *CFTR* analysis should be offered to the female partner, followed by information about options of prenatal or preimplantation genetic diagnostics if both partners are carriers of at least one severe *CFTR* mutation.

If *CFTR* mutations are excluded, genetic analysis should be extended to the X-chromosomal gene *ADGRG2* in males with obstructive azoospermia. Pathogenic variants in *ADGRG2* account for around 2 % of CBAVD cases [20].

### Genetic diagnostics in men with specific sperm defects

Male infertility is not only the consequence of a decreased sperm count but may as well result from aberrantly shaped or non-motile sperm (terato- and asthenozoospermia). For isolated teratozoospermia, genetic testing is useful if the sperm have specific and consistent defects such as globo- or macrozoospermia (round-headed sperm or enlarged sperm heads). In these cases, gene panel analysis can be offered. For the respective genes, see Table 2.

Patients with asthenozoospermia may carry variants in genes responsible for the assembly and/or function of the sperm flagellum. Such variants may also induce an aberrant sperm morphology called asthenoteratozoospermia, a combination of both aberrant shape and motility; a subform of asthenoteratozoospermia exhibits multiple morphological abnormalities of the sperm flagella (MMAF). In contrast, if only sperm motility is affected and accompanied by a patient history of recurrent sinusitis, bronchitis, pneumonia or otitis media, primary ciliary dyskinesia (PCD) can be suspected. A subform of PCD is Kartagener syndrome, with a situs inversus found in addition to the mentioned symptoms. The phenotypic and genetic spectrum of MMAF and PCD is diverse, where both are associated with large numbers of genes, and partially overlapping clinical features. To further complicate a preliminary diagnosis based on classic semen parameters, both MMAF and PCD can be accompanied by reduced sperm counts resulting in OAT. Thus, at least when morphology and/or motility are significantly reduced, a gene panel analysis can be offered (see Table 2 for genes).

### Genetic diagnostics in men with hyper- and hypogonadotropic hypogonadism

A gene panel analysis can be offered to men with suspected CHH. However, currently no well-validated genotype–phenotype correlations are established that would change clinical care. Nevertheless, the genetic diagnosis may allow one to establish the recurrence risk for CHH in the family. Currently known genes are listed in Table 2.

**Table 2:** Selected monogenic disorders associated with infertility.

Phenotype	Genes
Obstructive azoospermia (OA)	*CFTR*, *ADGRG2* [30]
Non-obstructive azoospermia (NOA)	*AR*, *DMRT1*, *KLHL10*, *M1AP*, *NR5A1*, *SHOC1*, *STAG3*, *SYCE1*, *TEX11*, *TEX14*, *TEX15* [31, own data]
Globozoospermia	*DPY19L2*, *ZPBP*, *PICK1*, *SPATA16* [30]
Macrozoospermia	*AURKC* [30]
Multiple morphological abnormalities of the sperm flagella (MMAF)	*DNAH1*, *DNAH2*, *DNAH6*, *DNAH17*, *CFAP43*, *CFAP65*, *CFAP70*, *WDR66* (*CFAP251*), *FSIP2*, *CEP135*, *TTC21A*, *TTC29*, *SPEF2*, *CFAP69*, *QRICH2*, *AK7*, *ARMC2* [32]
Primary ciliary dyskinesia (PCD)	*CCNO*, *MCIDAS*, *CFAP298* (*C21ORF59*), *CFAP300* (*C11orf70*), *DNAAF1* (*LRRC50*), *DNAAF2* (*KTU*), *DNAAF3*, *DNAAF4* (*DYX1C1*), *DNAAF5* (*HEATR2*), *LRRC6*, *PIH1D3* (*DNAAF6*), *SPAG1*, *ZMYND10* (*DNAAF7*), *CCDC103*, *ARMC4*, *CCDC114*, *CCDC151*, *DNAH11*, *DNAH5*, *DNAH9*, *DNAI1*, *DNAI2*, *DNAL1*, *MNS1*, *NME8*, *TTC25*, *DNAH6*, *DNAH1*, *CCDC39*, *CCDC40*, *CCDC65* (*DRC2*), *DRC1*, *GAS8*, *DNAJB13*, *RSPH1*, *RSPH3*, *RSPH4A*, *RSPH9*, *STK36*, *HYDIN*, *GAS2L2*, *LRRC56* [33]
Congenital hypogonadotropic hypogonadism (CHH)	*FGFR1*, *CHD7*, *PROKR2*, *SEMA3A*, *ANOS1*, *GNRHR*, *SOX10*, *SEMA7A*, *KLB*, *SPRY4*, *PROK2*, *SOX2*, *DMXL2*, *IL17RD*, *KISS1R*, *GNRH1*, *TACR3*, *HESX1*, *DUSP6*, *FGF8*, *FGF17*, *POLR3A*, *POLR3B*, *PNPLA6*, *NSMF*, *TAC3*, *FLRT3*, *KISS1* [34]

## Genetic diagnostics in couples with normal clinical findings

### Karyotyping

In couples with unexplained infertility, meaning that clinical diagnostics did not detect a reason for this condition, karyotyping should be offered to both partners. Infertile couples are known to have an increased risk of carrying chromosomal aberrations, specifically a higher percentage of structural balanced chromosomal abnormalities such as translocations or inversions is seen, when compared with fertile couples. Most frequently, these couples exhibit balanced translocations; translocation carriers have an increased risk of children with unbalanced chromosomal aberrations, possibly leading to severe syndromic diseases including malformations and intellectual disability. For this reason, genetic counseling and prenatal or preimplantation genetic diagnostics must be offered to affected couples [10].

## Diagnostic work-up in recurrent miscarriage

In couples with recurrent miscarriages, the focus of medical examination lies on the female side, but both partners must undergo cytogenetic evaluation.

Congenital malformations of the uterus and acquired conditions like adhesions, polyps and myoma may be associated with a slightly increased miscarriage risk and warrant ultrasound examinations or hysteroscopy. Endocrine dysfunctions that can cause miscarriages are manifesting hyper- or hypothyroidism, while latent hypothyroidism (increased TSH, normal T3 and T4 values) is not an established risk factor for miscarriages. There is no evidence that inherited thrombophilia causes first trimester miscarriages; as such, treatment with neither heparin nor ASS is recommended to avoid a further miscarriage. In contrast to previous clinical practice, a thrombophilia screening (antithrombin or protein C/protein S activity, genetic variants factor II/prothrombin G20210A or factor V Leiden) is no longer recommended. However, since an increased risk for thrombosis can be relevant for future pregnancy management, thrombophilia screening is indicated if the woman reports thromboembolic events in her own or in her family’s medical history. Immunological factors as risk factors for miscarriages are largely restricted to antiphospholipid syndrome (APS), which should be excluded by means of physical examination and laboratory values (elevated antibodies against anticardiolipin or anti-β2-glycoprotein, lupus anticoagulants).

**Table 3:** Risk of a balanced chromosomal aberration in maternal age groups in relation to family history of recurrent miscarriage and number of previous miscarriages (adapted from Franssen et al., 2005 [35]).

	At least two miscarriages in family members		Risk of balanced chromosomal aberration (%) according to no. of previous miscarriages	
		
				
Maternal age (years)	Sibling	Parent	≥3	2
<23	+	+	10.2	7.3
	+	–	7.3	5.2
	–	+	5.7	4.0
	–	–	4.1	2.8
23–33	+	+	10.0	7.2
	+	–	7.2	5.1
	–	+	5.7	4.0
	–	–	4.0	2.8
34–36	+	+	5.8	4.1
	+	–	4.1	2.9
	–	+	3.2	2.2
	–	–	2.2	1.6
37–38	+	+	4.0	2.8
	+	–	2.8	2.0
	–	+	2.2	1.5
	–	–	1.5	1.1
≥39	+	+	1.8	1.2
	+	–	1.3	0.9
	–	+	1.0	0.7
	–	–	0.7	0.5

The risk of recurrent miscarriages is correlated with maternal age, while paternal age does not play a major role. Numerical chromosomal abnormalities are responsible for 60–70 % of first trimester miscarriages. In 4–5 % of couples with at least two miscarriages, one partner carries a balanced structural chromosome abnormality, compared with 0.7 % in the normal population and 1 % in couples after one miscarriage [21]. The probability increases with the number of family members with recurrent miscarriages (Table 3). For this reason, conventional karyotyping in both partners is recommended, since balanced translocations or inversions bear the risk of an unbalanced chromosomal aberration in offspring (see above). The molecular (cytogenetic) diagnosis of chromosomal copy number variations (CNVs) in material obtained from the miscarriage (denoted as product of pregnancy) is technically feasible with to-date methods, but not widespread practice yet and, thus, data are lacking to formulate general advice. But these recommendations may change in the future. There are many genetic and environmental factors that contribute to early pregnancy loss. Currently, with the exception of familial chromosomal aberrations, routine investigation for monogenic factors is not applicable.

## Risk of birth defects in infants conceived by ART

The average risk of birth defects for naturally conceived singleton infants is approximately 3 % [22]. As infertile couples have an increased risk of carrying chromosomal aberrations, the risk that their offspring will have birth defects is also increased. Moreover, several studies have suggested that infants conceived by ART and, in particular, intracytoplasmic sperm injection (ICSI) might have a further increased risk: A recent large and well-controlled study found an 18 % increased risk of a major non-chromosomal birth defect in singleton infants conceived with ART by *in vitro* fertilization (IVF) (without ICSI) and a 30–42 % increased risk with ICSI [23]. It has also been reported that the risk for some imprinting disorders, such as Beckwith–Wiedemann syndrome, might be slightly increased with ICSI [24]. Couples must be made aware of these increased risks prior to ART.

## Outlook

Most couples with non-syndromic infertility remain without a causal diagnosis [25]. While various novel monogenic causes for male infertility have been described in the last few years, the majority of these genes warrant further validation. Thus far, most of these genes have a low level of evidence for a gene–disease association. Furthermore, prospective studies including clinical predictions, e. g., for chances of TESE, are still mostly lacking, and such sparse data complicate the interpretation of detected variants in these genes. Still, a subset of genes can now be offered for analysis in the case of NOA and cryptozoospermia. In a pilot scheme of prospective exome sequencing in more than 800 NOA men, around 3 % of additional diagnostic mutations were identified (authors’ own data, manuscript in preparation). Some of these findings can be immediately relevant for infertility counseling/treatment, as they may indicate the success of TESE.

Evidence is accumulating that children, especially boys, conceived by ICSI may have reduced fertility when becoming adults [26] – which may not be surprising to the genetic community. Also, an association between male infertility and chronic disease and mortality has been described, which should be assessed more systematically [27], [28]. Lastly, advanced paternal age also poses risks. While the overall risk to offspring is still low (less than 0.5 %), autosomal dominant *de novo* mutations of clinically significant disorders are increased by a factor of 10 in fathers aged more than 50 compared with the general population [29]. However, it would be premature to address these issues during routine genetic counseling, as either they are not important in the context of family planning, or data are not adequate for drawing reliable conclusions or insufficient to estimate specific risks. Still, the authors advise to closely monitor these topics as they may become relevant in the future with respect to the broader “reproductive health.”

## Conclusions for clinical practice

– Genetic disorders are the reason for 5–10 % of female and 5–20 % of male infertility.
– In 10–13 % of women with ovarian dysfunction (hypergonadotropic hypogonadism), a gonosomal aberration (Turner syndrome, trisomy X syndrome) can be diagnosed.
– In women with primary or secondary ovarian insufficiency, analysis of the *FMR1* premutation must be performed.
– Male and female patients presenting with hypogonadotropic hypogonadism can be analyzed by a gene panel (CHH genes).
– In male infertility, semen and hormone parameters may point toward a specific genetic diagnosis.
– The most important genetic reason for azoospermia and male hypergonadotropic hypogonadism is Klinefelter syndrome.
– In male patients with non-obstructive azoospermia, chromosomal analysis and screening for AZF microdeletions must be performed. These analyses should also be performed in men with severe oligozoospermia (<5×10^6^ sperm/ml).
– *CFTR* analysis must be performed in men with signs of obstructive azoospermia. This analysis should be extended to patients with unexplained azoospermia without hypogonadism.
– Genetic testing can be performed in specific sperm morphology/motility defects such as globo-/macrozoospermia, MMAF or PCD.
– Infertile couples have an increased risk for chromosomal aberrations, even if there are no abnormal clinical findings. Therefore, a chromosomal analysis should be performed in both partners prior to undergoing ART.
– In couples with recurrent miscarriages, karyotyping must be performed in order to detect balanced structural chromosomal aberrations.
